# Post-Marketing Safety Surveillance of HPV Vaccines in Anhui Province, China, 2017–2024

**DOI:** 10.3390/vaccines13080846

**Published:** 2025-08-09

**Authors:** Fanya Meng, Wenyi Hu, Binbing Wang, Xianwei Luo, Haiyang Xu, Jiangshun Wan, Wenqing Xue, Ying Su, Yong Sun, Jiabing Wu

**Affiliations:** 1 Anhui Provincial Center for Disease Control and Prevention, Hefei 230601, China; mfy@ahcdc.com.cn (F.M.); wbb@ahcdc.com.cn (B.W.); lxw@ahcdc.com.cn (X.L.); xuewenqin@ahcdc.com.cn (W.X.); sy@ahcdc.com.cn (Y.S.); sunyong@ahcdc.com.cn (Y.S.); 2 School of Public Health, Bengbu Medical University, Bengbu 233030, China; 18360708595@163.com; 3 Fuyan Center for Disease Control and Prevention, Fuyang 236000, China; fysjmk@163.com; 4 Anqin Center for Disease Control and Prevention, Anqing 246003, China; anqingepi@163.com

**Keywords:** human papillomavirus vaccination, cervical cancer, adverse events following immunization, vaccine safety, China

## Abstract

**Background:** The Human Papillomavirus (HPV) vaccine has been globally implemented to prevent HPV-associated diseases. Since its introduction in China in 2017, this vaccine has substantially reduced the burden of HPV-related health conditions. As more people get vaccinated these days, ongoing safety monitoring and evaluation have become critical. This helps to safeguard public trust in immunization programs and ensures the sustainability of immunization programs. **Methods:** Suspected adverse events following immunization (AEFIs) associated with HPV vaccination from 2017 to 2024 were extracted from the Chinese National Immunization Information System (CNIIS). Data on the number of HPV vaccine doses given came from the Immunization Planning Information Management System of Anhui Province. Descriptive statistical methods examined the distribution characteristics of AEFIs, and chi-square tests assessed differences in incidence rates. **Results:** From 2017 to 2024, the vaccine safety surveillance system in Anhui Province monitored a total of 1149 reports of AEFIs associated with the HPV vaccine. The reported overall rate of AEFIs was 16.32 per 100,000 doses. Specifically, the rates of common adverse reactions were 15.15 per 100,000 doses, while the rates for rare adverse reactions were 0.85 per 100,000. Among the common adverse reactions, the incidence rates of injection-site redness and swelling (diameter >5.0 cm), induration (diameter >5.0 cm), and fever (axillary temperature ≥38.6 °C) were 0.60, 0.33, and 1.34 per 100,000 doses, respectively. For rare adverse reactions, the reported incidence rates of allergic rash, allergic urticaria, and aseptic abscess were 0.50, 0.09, and 0.03 per 100,000 doses, respectively. Most AEFIs occurred within 24 h post-vaccination. **Conclusions:** The overall reported incidence of AEFIs following HPV vaccination in Anhui Province from 2017 to 2024 was low, with serious rare adverse reaction occurring infrequently. These findings suggest that the HPV vaccine has a favorable safety profile.

## 1. Introduction

Human Papillomavirus (HPV) infection is one of the most common sexually transmitted diseases worldwide and the main cause of cervical cancer. Cervical cancer is the fourth most common malignant tumor among women globally, following breast, lung, and colorectal cancers, posing a serious threat to women’s health [1]. According to GLOBOCAN 2022 data, there were approximately 348,189 cervical cancer deaths and 661,021 new cases in 2022 [2]. Globally, about 94% of cervical cancer deaths worldwide take place in low- and middle-income countries, with Asia bearing the heaviest disease burden, making it the major region for cervical cancer incidence and mortality. In China, there were 150,659 new cases of cervical cancer and 55,694 deaths, and the incidence and mortality rates of cervical cancer were 13.8 and 4.5 per 100,000 [2,3]. The HPV vaccine is the world’s first vaccine for cancer prevention, marking the first time that mankind has attempted to eradicate a kind of cancer through a vaccine. Since its introduction to the Chinese market in 2017, the use of the HPV vaccine, as a crucial defense against cervical cancer and other HPV-related diseases, has been gradually expanded, with the number of administered doses increasing annually, gradually being extended to age-appropriate female populations. HPV vaccines are not currently included in the National Immunization Program (NIP) in China, and the cost is primarily borne by vaccine recipients. As the HPV vaccine becomes widely implemented in China, monitoring suspected Adverse Events Following Immunization (AEFIs) has become crucial. Although pre-licensure clinical trials have demonstrated the vaccine’s safety, post-marketing surveillance data on AEFIs require further in-depth analysis and continuous monitoring. The purpose of this research was to analyze the post-marketing AEFI monitoring data of the HPV vaccine in Anhui Province from 2017 to 2024, ensuring the safety of the HPV vaccine by continuously evaluating adverse reactions after vaccination.

## 2. Materials and Methods

### 2.1. Data Collection

Data regarding AEFIs associated with the HPV vaccine in Anhui Province from 2017 to 2024 were obtained using the AEFI surveillance module of the China Disease Control and Prevention Information System, and the number of HPV vaccination doses during the same period. Descriptive analyses and chi-square tests were performed to monitor the characteristics and reported incidence rates of AEFIs associated with HPV vaccines.

### 2.2. Types of HPV Vaccines and Vaccination Procedures

Five HPV vaccinations are presently available in China to prevent HPV infection. These include three 2-valent vaccines targeting HPV types 16 and 18: the 2-valent HPV vaccine (adsorbed), the 2-valent HPV vaccine (*E. coli*), and the 2-valent HPV vaccine (*P. pastoris*). Additionally, there is one 4-valent vaccine (*S. cerevisiae*) targeting HPV types 6, 11, 16, and 18, and one 9-valent vaccine (*S. cerevisiae*) covering HPV types 6, 11, 16, 18, 31, 33, 45, 52, and 58 [4]. All five HPV vaccines have been used in completed clinical trials, with studies confirming their good tolerability and immunogenicity. Most adverse events reported were mild reactions [5,6]. The HPV vaccine is appropriate for women between the ages of 9 and 45 years. The 2-valent HPV vaccines require three doses of 0.5 mL each, administered at 0, 1, and 6 months. Both the 4-valent and 9-valent HPV vaccines follow a three-dose schedule (0.5 mL per dose) at 0, 2, and 6 months. All types of HPV vaccine are administered by intramuscular injection into the deltoid muscle of the arm.

### 2.3. Definition and Categorization of AEFIs

An AEFI refers to any suspected adverse reactions or events that occur after vaccination and may be associated with the vaccination. According to the National Program for Monitoring Adverse Events Following Immunization [7], AEFIs are classified into adverse reactions, psychogenic reactions, coincidental events, vaccination accidents, and vaccine quality incidents. Adverse reactions are divided into common adverse reactions and rare adverse reactions. Common adverse reactions are those that occur after vaccination due to the inherent properties of the vaccine, causing temporary physiological disturbances in the body. These mainly consist of fever and localized redness and swelling, while additional symptoms like weariness, loss of appetite, and overall discomfort may also be present. Rare adverse reactions refer to reactions caused by a qualified vaccine during or after a standard vaccination process, which leads to damage to the body’s tissues, organs, and functions, with no fault from any party involved. Psychogenic reactions are those triggered by psychological or mental factors in the recipient after vaccination, unrelated to the vaccine ingredients. Coincidental events refer to situations when the immunization is administered while the recipient is in the prodromal or incubation stage of an illness, and the disease coincidentally manifests after vaccination. These illnesses are usually caused by factors such as infections and are not due to the vaccine’s characteristics. The classification of AEFI severity (mild/moderate/severe) and the determination of serious adverse events were based on a retrospective clinical evaluation using complete medical records, following the National AEFI Surveillance Guidelines. Serious adverse events include death, life-threatening conditions, hospitalization or prolonged hospitalization, persistent or significant disability, congenital anomalies or birth defects (if vaccination occurred during pregnancy), and other events that may lead to such outcomes without medical intervention. Typically, these cases require hospital treatment, including serious illnesses requiring clinical care. Examples include anaphylactic shock, laryngeal edema, Henoch–Schönlein purpura (HSP), Guillain–Barré syndrome (GBS), encephalopathy, and meningitis that are suspected to be related to the vaccine.

### 2.4. Monitoring Method

The safety monitoring of HPV vaccines in this study was mainly based on a passive surveillance system, namely the China National AEFI Surveillance System (CNAEFIS). Established in 2005, the CNAEFIS is a nationwide post-marketing passive vaccine safety monitoring system. It relies on the reporting of AEFIs to detect known reactions, identify unknown ones, and analyze possible risk factors. Responsible reporters include medical institutions, vaccination units, disease control agencies, adverse drug reaction monitoring centers, vaccine manufacturers and distributors, and related personnel. The system operates under a local management model. When a suspected AEFI occurs, it must be reported promptly to the county-level health and drug regulatory departments. Most reports should be submitted within 48 h using the AEFI case report form. In the case of death, severe disability, clustering of events, or socially significant reactions, the event must be reported within 2 h by phone or other urgent means. Once verified, the report is submitted through the National Immunization Information System. All levels of relevant agencies monitor the data in real time. For fatal or clustered AEFIs, additional reporting procedures must follow the national regulations on public health emergencies.

### 2.5. Statistical Analysis

Data were managed and analyzed using Microsoft Office Excel (version 2021, Microsoft, Washington, DC, USA) and SPSS (version 27.0, IBM Corporation, Armonk, NY, USA). The distribution, characterization, and reported incidence of AEFIs were explained using descriptive statistics. The chi-square test was employed to compare the reported incidence of AEFIs. The age was calculated based on the interval between the date of birth and the date of the reaction occurrence. The incidence rate of reported AEFIs was calculated using the following formula: number of reported cases of AEFIs with HPV vaccine/number of HPV vaccine doses administered × 100,000 doses.

## 3. Results

### 3.1. HPV Vaccines Administration

**Figure 1:**
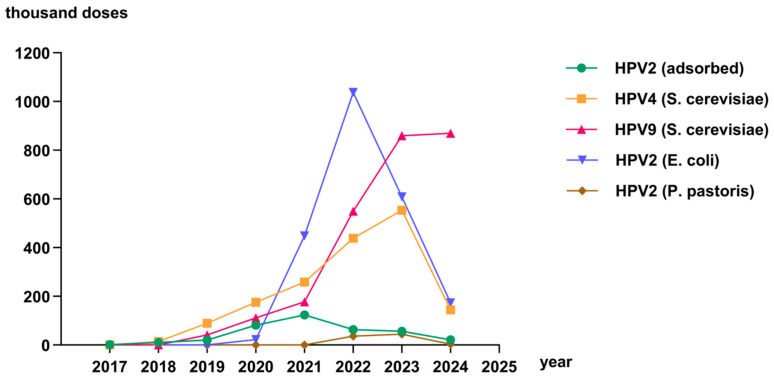
The number of doses of various types of HPV vaccines administered in Anhui Province, China, 2017–2024.

In this study, the HPV vaccination rate in Anhui Province from 2017 to 2024 was estimated based on the total number of females aged 9–45 years from the Anhui Provincial Population Census Yearbook 2020 [8] as the denominator. The HPV vaccination rate in Anhui Province was below 1% from 2017 to 2019. In 2020, 2021, 2022, 2023, and 2024, the vaccination rates were 1.55%, 4.13%, 8.03%, 8.34%, and 5.00%, respectively. Before 2019, the HPV vaccination rate in Anhui Province increased slowly. However, there was a significant rise after 2019, reaching its peak in 2023 at 8.34%. Between 1 January 2017 and 31 December 2024, a total of 7,038,333 doses of HPV vaccine were administered in 16 cities in Anhui Province. The two-valent HPV vaccine (adsorbed) had the highest number of doses administered in 2021, followed by a decline thereafter. The number of doses administered for the two-valent HPV vaccine (*E. coli*) began to increase significantly in 2021, peaked in 2022, and then declined in the following two years, but still remained high. The two-valent HPV vaccine (*P. pastoris*) had a relatively small number of doses due to its late introduction to the market. The number of four-valent HPV vaccines (*S. cerevisiae*) increased year by year from 2017 to 2022, and decreased in 2023 and 2024. The number of doses administered for the nine-valent HPV vaccine (*S. cerevisiae*) had seen a rapid increase since 2018, and it has become the most widely administered HPV vaccine type (Figure 1).

### 3.2. Classification of AEFIs

Between 2017 and 2024, about 7,038,333 doses of the HPV vaccination were provided in Anhui Province, and 1149 HPV vaccine-related AEFIs were reported, with a reported incidence of AEFIs of 16.32 per 100,000 doses. These cases were classified based on the cause of event occurrence, with 1066 cases of common adverse reactions with a reported incidence of 15.15 per 100,000, 60 cases of rare adverse reactions with a reported incidence of 0.85 per 100,000, 12 cases of psychogenic reactions with a reported incidence of 0.17 per 100,000, 10 cases of coincidental events with a reported incidence of 0.14 per 100,000, and 1 case of a pending event, with a reported incidence of 0.01 per 100,000; no vaccination accidents or vaccine quality incidents related to reaction events were reported (Table 1). The incidence rates of common adverse reactions showed statistically significant variations across different HPV vaccine types (*χ*^2^ = 63.06, *p* < 0.05), with no statistically significant difference in the reported incidence of rare adverse reactions and psychogenic reactions (*χ*^2^ = 8.63, 3.72, *p* > 0.05), and no statistically significant difference in the reported incidence of incidental reactions to the four-valent vaccine (*S. cerevisiae*) and nine-valent vaccine (*S. cerevisiae*) (*χ*^2^ = 0.07, *p* > 0.05).

### 3.3. Distributional Characteristics of AEFIs

A total of 1149 cases of AEFIs caused by HPV vaccination were reported in Anhui Province from 2017 to 2024. In terms of the distribution of AEFI reports by age group, the largest number of cases occurred in those aged ≥27 years, totaling 741 cases, accounting for 64.49%. Regarding the timing of reports, most AEFI cases were reported in the second quarter, totaling 312 cases, which made up 27.15% of all AEFI cases. In terms of vaccination doses, adverse reactions after HPV vaccination mainly occurred following the initial dose of vaccination, totaling 579 cases, accounting for 50.39% of all AEFI cases. In terms of the duration between vaccination and the manifestation of symptoms, the majority of AEFIs happened within one day of vaccination, with 1000 cases, accounting for 87.03% of the total. In terms of whether they were serious AEFIs, most of them were non-serious AEFIs, with 1134 cases, making up 98.69%. Severe AEFIs were rare, with only 15 cases reported, accounting for only 1.31% (Table 2).

### 3.4. Clinical Diagnosis of Common Adverse Reactions

From 2017 to 2024, a total of 1066 cases of common adverse reactions were reported in Anhui Province. Among them, there were 414 cases of fever, 562 cases of local redness and swelling, and 389 cases of induration at the injection site; the reported incidence rates were 5.88, 7.98, and 5.53 per 100,000 doses, respectively. The differences in the reported incidence of fever, local redness and swelling, and induration among the different types of HPV vaccine general reactions were statistically significant (*χ*^2^ = 54.55, 25.51, and 15.87, *p* < 0.05). Additionally, there were 106 cases of other general reactions, with a reported incidence of 1.51 per 100,000. Among them, 94 cases of fever (axillary temperature ≥ 38.6 °C), 42 cases of redness and swelling (diameter > 5.0 cm), and 23 cases of induration (diameter > 5.0 cm) were reported, with reported incidence rates of 1.34, 0.6, and 0.33 per 100,000, respectively (Table 3). The difference in the reported incidence of clinical manifestations of other common adverse reactions for different types of HPV vaccines was statistically significant (*χ*^2^ = 13.85, *p* < 0.05).

### 3.5. Clinical Diagnosis of Rare Adverse Reactions

Between 2017 and 2024, a total of 60 cases of rare adverse reactions were reported in Anhui Province. Among these allergic reactions, there were 35 cases of allergic rash, 6 cases of allergic urticaria, 2 cases of anaphylactic shock, 1 case of allergic maculopapular rash, and 5 cases of other allergic reactions. The reported incidence rates were 0.5, 0.09, 0.03, 0.01, and 0.07 per 100,000 doses, respectively. The incidence rates of allergic responses for the various HPV vaccination types did not differ statistically significantly (*χ*^2^ = 5.56, *p* > 0.05). For nervous system diseases, there were two cases of GBS, one case of meningitis, and one case of brachial plexus neuritis (BPN), with reported incidence rates of 0.03, 0.01, and 0.01 per 100,000, respectively. The differences in the incidence rates of nervous system diseases among different types of HPV vaccines were not statistically significant (*χ*^2^ = 6.86, *p* > 0.05). In addition, there was one event of HSP, two events of an aseptic abscess, and four events of other rare adverse reactions, with reported incidence rates of 0.01, 0.03, and 0.06 per 100,000, respectively (Table 3).

## 4. Discussion

Cervical cancer poses a major public health threat to global female health, which is closely linked to persistent high-risk HPV infection. A crucial preventive measure against cervical cancer is HPV vaccination. With the acceleration of the application process of the HPV vaccine in China, the number of people receiving HPV vaccine has increased significantly, and the importance of immunization for the prevention of cervical cancer has been gradually highlighted. According to a Swedish research cohort, girls under the age of 17 years who were vaccinated against HPV had an 88% lower chance of having cervical cancer as adults than those who were not. Among women aged 17 to 30 years, the incidence of cervical cancer was reduced by 53% in those who were vaccinated [9], emphasizing the strong protective effect of vaccination during adolescence. Additionally, a study published in The Lancet predicted that if the HPV vaccination rate reaches 80% to 100% among the target population, combined with high-quality cervical cancer screening at ages 35 and 45 years, the global goal of eliminating cervical cancer could be achieved between 2055 and 2059 [10]. Since the introduction of the HPV vaccine in China in 2017, the vaccination rate of the female population aged 9–45 years has exhibited a rising trend year by year, but as of 2022, the rate of uptake of the first dose was only 10.15%, and the full-course completion rate was just 6.21% [11], which is significantly lower than the global average [12]. This indicates that China’s HPV vaccination rate still needs to be improved. In Anhui Province, the HPV vaccination rate also increased each year from 2017 to 2024, rising from less than 1% to 8.34%. These data indicate that Anhui Province has made significant progress in increasing HPV vaccination rates, but it is below the national average and far from the level needed to achieve herd immunity against cervical cancer, and continued efforts will be needed to reach higher vaccination targets in the future. According to a cross-sectional survey of 4220 women in college in 2019, 11% of respondents self-reported that they had already received the HPV vaccine, while 53.5% of respondents who were not vaccinated expressed a willingness to be vaccinated. The survey showed that the social-economic level, the level of knowledge about HPV vaccine, the perception of benefits and risks of the vaccine, and sexual behavior were the main factors influencing the willingness to vaccinate. These factors also affected the actual vaccination rate to some extent [13]. Another study focused on women aged 18 to 45 years revealed that, despite a high willingness to be vaccinated, only 3% of participants had received the HPV vaccine within three years of its approval. This was due to a lack of knowledge, safety concerns, and the great expense of the vaccination. The current low vaccination rate is also closely related to the fact that the national immunization program does not yet include the HPV vaccine, and the economic burden of the vaccine [14]. Despite the challenge of low vaccination rates, a number of provinces have taken proactive measures to gradually explore various ways to increase vaccination coverage, especially making sure women who qualify in poorer areas can access the vaccine. For example, since 2021, some provinces have included HPV vaccines in government livelihood programs, providing free HPV immunization services to local girls of the appropriate age of between 13 and 14 years old through the financial purchase of services and flat-rate subsidies [15]. In Anhui Province, HPV vaccination is currently based on a self-paid and voluntary model. Only in some pilot areas is the domestic bivalent HPV vaccine provided free of charge to girls aged 13–14 years. To steadily advance this public health initiative, Anhui Province is summarizing its pilot experience and plans to launch a free HPV vaccination program for eligible girls as a provincial public health project in 2025. This policy is expected to significantly increase HPV vaccine coverage among adolescents and strengthen the primary prevention of cervical cancer. With growing local government support and improvements in national immunization policies, the equity and accessibility of HPV vaccination are likely to improve further. These efforts will help reduce the economic burden and ensure timely access to vaccination for eligible age groups. With the increasing number of HPV vaccinations, its post-marketing safety monitoring is crucial. In this study, we analyzed the AEFI reports after HPV vaccination in Anhui Province from 2017 to 2024, and the results showed that the total reported incidence of HPV-vaccine-related AEFIs was 16.32 per 100,000 doses, of which the proportion of serious adverse reactions was only 1.31%, and the total reported incidence was significantly lower than the national average [16] but higher than that in Guangdong [17], Hebei [18], Jiangxi [19] provinces. These differences may be due to several factors, including vaccination rates, surveillance efforts, and reporting awareness in different regions. Differences in HPV vaccination coverage and reporting systems across provinces may affect the number and types of AEFI reports, reflecting variations in the ability to report and monitor AEFIs. In the future, efforts are needed to improve surveillance sensitivity and data quality at the local level, building on the national unified system, in order to reduce regional differences. Other factors, such as demographic characteristics, vaccine availability, and utilization, might also affect the reported incidence. For the nine-valent vaccine, the reported incidence in Anhui Province was 19.36 per 100,000 doses. This rate was lower than that reported by the U.S. Vaccine Adverse Event Reporting System (VAERS) [20], but higher than the rate in the Puglia region of Italy [21]. In contrast, the reported incidence for the four-valent vaccine was 12.36 per 100,000 doses, which was lower than the reported incidence in Australia [22]. These differences may come from variations in national surveillance systems, vaccination strategies, age distribution of the vaccinated population, vaccine quality standards, and the sensitivity of surveillance systems.

The reported incidence of common adverse reactions in this study was 15.15 per 100,000. These reactions were mainly mild, such as fever, redness and swelling, and induration. The reported incidence of rare adverse reactions was 0.85 per 100,000, which is much lower than the reported incidence of both common adverse reactions and rare adverse reactions in studies from Spain [23] and Australia [24]. Most AEFI reports were mainly concentrated within 24 h after vaccination, which was consistent with the results of the Jiangxi [19] and Gansu [25] province studies. Regarding the age of those with AEFI reports, Anhui Province had the highest number of AEFI cases in those aged ≥27 years, which was consistent with the results of the Zhengzhou study [26]. This may be due to higher awareness of cervical cancer prevention and a greater willingness to get vaccinated in this age group. The age expansion policy for the nine-valent vaccine and relatively stable economic resources in this group may also play a role. The highest number of AEFI cases for the nine-valent vaccine was found in those aged 18–26 years. This may be because this age group received the highest number of doses and represented the majority of nine-valent-vaccine recipients. During the study period, HPV vaccination in the region mainly targeted females, which is why the dataset focuses on female recipients. However, the public health value of vaccinating males has been widely recognized globally. In some areas of China, pilot programs for male HPV vaccination have been launched. In the future, as the demand for male vaccination increases, we recommend conducting targeted AEFI surveillance to assess vaccine safety among different gender groups, thereby providing data support for comprehensive HPV prevention strategies. Regarding the quarter of reporting, the highest number of AEFI cases occurred in the second quarter. The reports for different types of vaccines varied across the quarters, which could be influenced by vaccination schedules and seasonal effects on the immunological system of the body. In addition, the first dose of vaccination was associated with the highest number of reported AEFI cases. This could be due to a stronger immune response upon initial exposure to vaccine antigens, followed by increased tolerance to subsequent doses, leading to a lower incidence of adverse reactions. It is noteworthy that 12 cases of psychogenic reactions were reported in this study, and the occurrence of this condition may be related to the fact that the vaccinees did not eat, or developed nervousness. Therefore, it is recommended to remain seated during vaccination and avoid vaccination on an empty stomach. Among the rare adverse reactions, allergic reactions and nervous system diseases were the main types. Of the allergic reactions, allergic rash was the most common, with a reported incidence of 0.5 per 100,000 doses. Although the overall incidence of allergic reactions was low, severe allergic reactions like anaphylactic shock still require a high degree of vigilance. Individuals with a history of allergies, particularly to vaccine components, should undergo rigorous evaluation with careful decision-making before HPV vaccination, and they should be closely monitored afterward. Among the neurologic diseases, GBS was reported at an incidence of 0.03 per 100,000 doses, and meningitis and brachial plexus neuritis at 0.01 per 100,000. The incidence of these nervous system diseases was extremely low, and this study showed no causal relationship between the cases of meningitis and vaccination, while the causal relationship between GBS or brachial plexus neuritis and vaccination was unclear. The current research indicated that the risk of serious neurologic diseases such as GBS was not significantly increased after HPV vaccination [27]. In addition, Henoch–Schönlein purpura, aseptic abscesses, and other rare adverse reactions were also reported in this study. Although these rare adverse reactions were relatively rare, they still need to be focused on, especially when related symptoms occur after vaccination. Most of the AEFIs in this study were in the non-severe category and typically resolved with appropriate management or self-recovery, supporting the HPV vaccine’s positive safety profile even further. Although the proportion of severe AEFIs is low, the potential serious health consequences for individuals should not be overlooked. Therefore, continuous monitoring, accurate diagnosis, and prompt management of severe AEFIs are crucial. In the future, the AEFI surveillance system should be further improved to better detect and report severe cases. This will help to ensure timely detection and effective intervention. For severe AEFI cases that have already occurred, in-depth investigation and analysis are needed to identify the causes and risk factors. This will enable the implementation of targeted preventive measures in future vaccination efforts, ultimately reducing the risk of severe AEFIs.

Overall, surveillance of AEFIs after HPV vaccination in Anhui Province from 2017 to 2024 showed that the vaccine is generally safe. However, vaccination monitoring still needs to be improved. Meanwhile, in order to increase vaccination rates and improve strategies, it is essential to continue promoting the HPV vaccine and carrying out public education about the HPV vaccine. Tailored health communication strategies should be used for different age groups. For example, for adolescents, it is important to raise parents’ awareness and understanding of HPV vaccination. For adult women, the focus should be on educating them about the importance of timely vaccination and its protective benefits. These efforts will help to increase public awareness of the HPV vaccine and reduce vaccine hesitancy. Meanwhile, active surveillance of AEFIs should be strengthened to promptly identify any potential safety signals related to the vaccine. Future studies ought to focus on the occurrence of AEFIs with different types of HPV vaccines, especially after long-term vaccination, to ensure the vaccine’s sustained safety and effectiveness. Furthermore, it is also advised that the provincial immunization program incorporate the HPV vaccine. To boost vaccination rates, policies including government procurement and subsidies can be used to lower vaccination costs. This will allow more women to benefit from the HPV vaccine, ultimately contributing to better cervical cancer prevention and long-term public health control.

## 5. Conclusions

The results of this study showed that the reported incidence of AEFIs after HPV vaccination in Anhui Province was low and mainly focused on common adverse reactions such as fever, redness and swelling, and induration. Serious adverse reactions were rare. AEFIs were most commonly reported after the first dose of the vaccine, with the majority occurring within the first 24 h after vaccination, and the vaccine has a good safety profile in general. However, there are inherent drawbacks of the passive surveillance system in this study, such as underreporting and regional differences in the level of investigation and diagnosis. As a result, the actual incidence of serious adverse reactions may be underestimated. In the future, it is necessary to further optimize the monitoring system by implementing targeted active surveillance, improving sensitivity and accuracy, and reducing underreporting. It is also recommended to strengthen the capacity of AEFI investigation and diagnosis, and improve the diagnostic level, and at the same time, strengthen the long-term safety tracking for the age-expanded vaccination recipients of the nine-valent vaccine, in order to provide a scientific basis for optimizing the vaccine strategy. It is only through the combined efforts of policy support and public participation that it will be possible to increase vaccination coverage while ensuring safety, ultimately working towards the public health goal of eliminating cervical cancer.

## Figures and Tables

**Table 1:** AEFI reported incidence rates per 100,000 doses of HPV vaccines in Anhui Province, China, 2017–2024.

Type	Common Adverse Reaction		Rare Adverse Reaction		Psychogenic Reaction		Coincidental Event		Pending Event		Total	
	No.	Incidence *	No.	Incidence	No.	Incidence	No.	Incidence	No.	Incidence	No.	Incidence
HPV2 (adsorbed)	79	20.87	7	1.85	0	0.00	0	0.00	0	0.00	86	22.71
HPV2 (*E. coli*)	298	13.01	12	0.52	2	0.09	0	0.00	0	0.00	312	13.62
HPV2 (*P. pastoris*)	29	34.18	0	0.00	0	0.00	0	0.00	0	0.00	29	34.18
HPV4 (*S. cerevisiae*)	193	11.52	19	1.13	2	0.12	3	0.18	0	0.00	217	12.95
HPV9 (*S. cerevisiae*)	467	17.91	22	0.84	8	0.31	7	0.27	1	0.04	505	19.36
Total	1066	15.15	60	0.85	12	0.17	10	0.14	1	0.01	1149	16.32

* Per 100,000 doses.

**Table 2:** Distribution characteristics of HPV vaccine AEFIs in Anhui Province, China, 2017–2024.

Characteristics	HPV2 (Adsorbed)		HPV2 (*E. coli*)		HPV2 (*P. pastoris*)		HPV4 (*S. cerevisiae*)		HPV9 (*S. cerevisiae*)		Total	
	No.	Proportion	No.	Proportion	No.	Proportion	No.	Proportion	No.	Proportion	No.	Proportion
		(%)		(%)		(%)		(%)		(%)		(%)
Age												
9–17	13	15.12	27	8.65	9	31.03	5	2.30	79	15.64	133	11.58
18–26	2	2.33	13	4.17	7	24.14	11	5.07	242	47.92	275	23.93
≥27	71	82.56	272	87.18	13	44.83	201	92.63	184	36.44	741	64.49
Quarter												
1	12	13.95	90	28.85	11	37.93	41	18.89	89	17.62	243	21.15
2	21	24.42	68	21.79	6	20.69	66	30.41	151	29.90	312	27.15
3	27	31.40	60	19.23	7	24.14	51	23.50	144	28.51	289	25.15
4	26	30.23	94	30.13	5	17.24	59	27.19	121	23.96	305	26.54
Dose												
1	41	47.67	132	42.31	16	55.17	104	47.93	286	56.63	579	50.39
2	23	26.74	83	26.60	3	10.34	58	26.73	104	20.59	271	23.59
3	22	25.58	97	31.09	10	34.48	55	25.35	115	22.77	299	26.02
Time Interval												
≤1	74	86.05	282	90.38	29	100.00	190	87.56	425	84.16	1000	87.03
2–7	8	9.30	26	8.33	0	0.00	19	8.76	44	8.71	97	8.44
8–14	2	2.33	3	0.96	0	0.00	3	1.38	8	1.58	16	1.39
≥15	2	2.33	1	0.32	0	0.00	5	2.30	28	5.54	36	3.13
Serious AEFI												
No	85	98.84	310	99.36	29	100.00	210	96.77	500	99.01	1134	98.69
Yes	1	1.16	2	0.64	0	0.00	7	3.23	5	0.99	15	1.31
Total	86	100.00	312	100.00	29	100.00	217	100.00	505	100.00	1149	100.00

**Table 3:** The incidence of adverse reactions after receiving different types of HPV vaccines in Anhui Province, China, 2017–2024.

Clinical Diagnosis	HPV2 (Adsorbed)		HPV2 (*E. coli*)		HPV2 (*P. pastoris*)		HPV4 (*S. cerevisiae*)		HPV9 (*S. cerevisiae*)		Total	
	No.	Incidence *	No.	Incidence	No.	Incidence	No.	Incidence	No.	Incidence	No.	Incidence
Common Adverse Reaction												
Fever (Axillary, °C)	43	11.36	96	4.19	11	12.96	72	4.30	192	7.36	414	5.88
37.1–37.5	19	5.02	38	1.66	6	7.07	38	2.27	73	2.80	174	2.47
37.6–38.5	16	4.23	31	1.35	1	1.18	21	1.25	77	2.95	146	2.07
≥38.6	8	2.11	27	1.18	4	4.71	13	0.78	42	1.61	94	1.34
Swelling (Diameter, cm)	34	8.98	175	7.64	18	21.21	109	6.51	226	8.66	562	7.98
≤2.5	10	2.64	92	4.02	11	12.96	52	3.10	112	4.29	277	3.94
2.6–5.0	18	4.75	69	3.01	6	7.07	53	3.16	97	3.72	243	3.45
>5.0	6	1.58	14	0.61	1	1.18	4	0.24	17	0.65	42	0.60
Induration (Diameter, cm)	29	7.66	130	5.67	11	12.96	73	4.36	146	5.60	389	5.53
≤2.5	9	2.38	65	2.84	7	8.25	37	2.21	83	3.18	201	2.86
2.6–5.0	18	4.75	57	2.49	4	4.71	33	1.97	53	2.03	165	2.34
>5.0	2	0.53	8	0.35	0	0.00	3	0.18	10	0.38	23	0.33
Other Common Adverse Reaction	3	0.79	26	1.13	2	2.36	18	1.07	57	2.19	106	1.51
Rare Adverse Reaction	7	1.85	12	0.52	0	0.00	19	1.13	22	0.84	60	0.85
Allergic Reaction	5	1.32	10	0.44	0	0.00	15	0.90	19	0.73	49	0.70
Neurologic diseases	1	0.26	1	0.04			2	0.12			4	0.06
Henoch–Schönlein purpura	0	0.00	0	0.00	0	0.00	0	0.00	1	0.04	1	0.01
Aseptic abscess	0	0.00	0	0.00	0	0.00	0	0.00	2	0.08	2	0.03
Others	1	0.26	1	0.04	0	0.00	2	0.12	0	0.00	4	0.06

* Per 100,000 doses.

## Data Availability

Data used for this study are not publicly available due to confidentiality restrictions.
